# Prevalence and factors associated with dry socket following routine dental extractions

**DOI:** 10.4317/medoral.26391

**Published:** 2024-01-30

**Authors:** Renan Bordini Cardoso, Vanessa Carvajal Soto, Ramon Cesar Godoy Gonçalves, Amanda Maria Pedroso, Roberto de Oliveira Jabur, Marcelo Carlos Bortoluzzi

**Affiliations:** 1 ORCID: 0000-0003-2832-9329. PhD Student. Oral and Maxillofacial Surgery Residency Program at University Hospital of Campos Gerais (HUCG), Ponta Grossa, Brazil; 2 ORCID:0000-0002-3372-8079. MSc Student. Dentistry Post-Graduate Program, State University of Ponta Grossa (UEPG), Ponta Grossa, Brazil; 3 ORCID: 0000-0002-7488-0771. PhD Student. Oral and Maxillofacial Surgery Residency Program at University Hospital of Campos Gerais (HUCG), Ponta Grossa, Brazil; 4 UGS Student. Dentistry Graduate Program, State University of Ponta Grossa (UEPG), Ponta Grossa, Brazil; 5 ORCID: 0000-0002-2026-1748. PhD. Professor at School of Dentistry, State University of Ponta Grossa (UEPG), Ponta Grossa, Brazil; 6 ORCID: 0000-0003-2756-5047. PhD. Professor at School of Dentistry, Dentistry Post-Graduate Program, State University of Ponta Grossa (UEPG), Ponta Grossa, Brazil

## Abstract

**Background:**

Dry socket (DS) or fibrinolytic osteitis is a relatively common complication that can occur following tooth extraction. This study aimed to determine the prevalence of DS and identify its associated predictive and mediating variables.

**Material and Methods:**

This study is classified as prospective observational, cross-sectional, and multicenter. Patients were consecutively selected in accordance with established criteria for tooth extraction. Data on patient demographics, surgical procedures and postoperative outcomes were collected. Nominal variables were analyzed using the Chi-Square Test, while associations involving ordinal values or considering counts or layers were examined using the Kendall's Tau-B Test or Mantel-Haenszel Test for trend. The GLM Mediation Model was employed to investigate potential mediation or indirect effects or potential underlying mechanisms of predictive variables on the development of DS. Two-tailed significance level of *p* ≤0.05 was considered statistically significant.

**Results:**

A total of 1,357 patients undergoing routine dental extractions were included. DS was observed in 13 patients (prevalence of 1%). DS was associated with younger patients (under 50 years old), longer procedures, and the presence of surgical accidents, but only when mediated by surgical complexity. Smoking, particularly in combination with complex surgeries and surgical accidents, was associated with DS. Postoperative pain for more than two days and reported at moderate to high levels, emerged as a potential warning sign for DS. The use of antibiotics was found to significantly reduce the risk of DS (RR reduction of 36% and absolute risk reduction of 0.63%).

**Conclusions:**

Routine dental extractions revealed a 1% prevalence of dry socket. The obtained results suggests that DS is a multifactorial condition influenced by various factors, including gender, age, smoking, antibiotic prescription and surgical factors such as length, technique and accidents, nevertheless, those associations were observed mainly considering the influence of one variable on another.

** Key words:**Dry socket, oral surgery, pain, dental extractions, antibiotics, tooth extraction, alveolar osteitis.

## Introduction

Dry socket (DS), also known as alveolar or fibrinolytic osteitis, is a relatively common complication that can occur following tooth extraction (1-9). It is characterized by the acute inflammation of the alveolar bone surrounding the extracted tooth, resulting in severe pain in the absence of infection or inflammation, typically appearing 2 to 4 days post-extraction. Clinical manifestations of DS encompass an empty socket with exposed bone, frequently containing food debris, mild swelling and redness of the gingiva, halitosis, and significant tenderness upon examination (3-6,8,10). The incidence of DS ranges from 1% to 5% for routine tooth extractions, but significantly higher rates have been reported for mandibular third molar extractions (1,4,5,7,9). As the occurrence of DS is more noTable in mandibular third molars, the majority of studies have focused on these procedures rather than regular or routine extractions, which are typically performed by general dentists.

The etiology of dry socket (DS) is not yet fully understood; however, the disintegration of the blood clot through fibrinolysis is widely accepted as the primary mechanism (2-5,11-14). Various factors have been associated with an increased risk of DS, although some of them remain controversial and may include difficult or traumatic extraction (6,15), poor oral hygiene (6,15), smoking (6-8,13,16), female gender (4,9), young patients (4), extraction site (15), previous DS (15) among other less frequently reported variables (12,13). Furthermore, a study using metagenomics to investigate the microbiome in DS sockets suggests that patients who develop DS may harbor a distinct and specific microbiota, indicating that bacteria may have a significant role in the etiology of DS as well (2).

The present study was designed as an observational, cross-sectional, and multicentric investigation with the primary objective of assessing the prevalence of DS following routine tooth extractions. In addition to estimating the prevalence, the study aimed to identify and examine the predictive and mediating variables that may be associated with the development of DS. By exploring a diverse population, the study sought to provide a comprehensive understanding of the occurrence of DS and its potential influencing factors. Thus, the study's primary hypothesis is to test whether any of the examined variables demonstrate a statistically significant impact on the occurrence of DS.

## Material and Methods

- Study design

The study was a prospective observational, cross-sectional, and multicenter investigation that aimed to determine the prevalence of dry socket (DS) and identify predictive and mediating variables or risk factors associated with its development. Ethical approval for the study was obtained from the University's Human Research Ethics Committees with the following registration numbers: UEPG-35061214.5.0000.0105 and UNOESC/HUST 250/2005. Informed consent was obtained from each participant prior to their inclusion in the study.

- Patient selection

Patients were consecutively selected in accordance with established criteria for tooth extraction. The inclusion criteria for the study were based on indications for tooth removal, such as irrecoverable teeth due to dental caries, periodontal disease, endodontics, prosthetic or orthodontic needs. Exclusion criteria included patients who had undergone surgery for impacted third molars, for extraction of deciduous teeth and lack of contact with the patient postoperatively or the inability to clinically check the occurrence of DS.

- Dental Extractions

Undergraduate dental students at the university's dental surgery clinics conducted all the exodontias. Stringent measures were taken to ensure strict control of microbiological contaminants throughout the procedures.

- Data Collection

A preoperative interview and anamnesis were firstly conducted. The postoperative outcomes of pain and inflammatory complications were assessed on the seventh postoperative day or whenever necessary, before or after this period. When faced with the presence of DS or any other surgical complication, the patients were followed up until the complete resolution of the problem. Telephone contact was used as a tool to collect symptoms from patients who were unable to return on the seventh day; however, no diagnosis was made under these circumstances.

- Criteria for Dry Socket (DS)

The criteria for DS was based on clinical condition (4,16). The clinical criteria for DS are as follows: (a) there is severe and persistent pain around the extraction site; (b) there is a partially or totally disintegrated blood clot with exposed alveolar bone; (c) halitosis is usually present. All diagnoses were always confirmed in a clinical setting by one oral and maxillofacial surgeon enrolled in the research. Doubts were resolved by consensus among the research team.

- Pain Evaluation

Pain levels were assessed on the seventh day after the surgery using a verbal category scale (VCS) with response options ranging from 0 to 3 (0 = none, 1 = mild, 2 = moderate, and 3 = severe). This method facilitated a qualitative classification of pain and allowed patients to evaluate the cognitive and emotional dimensions associated with their pain throughout the entire postoperative period. By capturing patients' memories, perceptions, and the impact of pain and injury, this comprehensive approach provided a deeper understanding beyond pain intensity alone and enables a more comprehensive understanding of the patient's pain experience, taking into account their subjective perception and overall evaluation of the pain's significance (17).

- Data analysis and Statistical Procedures

The JASP software (JASP Team, 2023, Version 0.18.1; University of Amsterdam, Netherlands) and/or the JAMOVI software (JAMOVI project, 2023, Version 2.4.9) were utilized for data analysis, employing descriptive and inferential methods. Two-tailed significance level of *p* ≤0.05 was considered statistically significant. The variables were categorized as continuous, ordinal, or nominal, and appropriate statistical tests were selected based on their characteristics, considering the distribution of the variables and checking the assumptions. Cases with missing sensitive data or lacking postoperative follow-up were excluded from the analysis. Surgical complexity was assessed by determining the necessity of flap elevation, osteotomy, and odontosection, resulting in an ordinal variable ranging from 0 to 3. Nominal variables were analyzed using the Chi-Square Test, while associations involving ordinal values or considering counts or layers were examined using the Kendall's Tau-B Test or Mantel-Haenszel Test for trend. The GLM Mediation Model (JAMOVI- Advanced Mediation Models) was employed to investigate potential mediation or indirect effects or potential underlying mechanisms of predictive variables on the development of DS.

## Results

The sample consisted of 1,357 routine dental extraction procedures. The majority of procedures were performed on male patients (714 or 52.6%), and the age ranged from 9 to 85 years (with a mean of 41 years, SD±15 years). One to three teeth were extracted per procedure (1 tooth: 1,023 surgeries; 2 teeth: 247 surgeries; 3 teeth: 87 surgeries). 20.4% of the patients reported having a systemic disease, while 22% were smokers.

Dry socket (DS) was observed in 13 patients (1%), with higher incidence among young female patients (average age of 35 years), although without statistically significant differences (proportion male-female 1:2.25; odds ratio for female =2.5). 5 DS cases were located in the anterior region of the maxilla, while 7 were found in the posterior region of the mandible, except for a single case that occurred in the canine area. 12 out of 13 cases of DS occurred in patients under 50 years old and the mean age of DS development was 34.7 (±11.1) years while for not having DS was 41.2 (±15.6). Epidemiological data of the study including the proportions and distributions of dental extractions and the development of dry socket can be seen on Table 1.

Regarding the surgical procedure, the average duration of the procedures was approximately 42 minutes (SD ± 27). As for the techniques employed, flap elevation was performed in 30% of cases [411], osteotomy in 12.7% [172], and odontosection in 13.5% [183]. The complexity of the procedure was determined by summing the requirements of the aforementioned techniques (as an ordinal measure ranging from 0 to 3). A higher value indicated a greater need for the utilization of all three techniques. The use of surgical flap, osteotomy and odontosection together was observed in 4.6% of the cases [62].

When the predictive variables were analyzed in isolation as a factor for DS development, it did not show statistically association with, sex, the presence of systemic disease, daily medication use (including contraceptives), contraceptives use (alone), or menstrual cycle influence. Habits such as tobacco, alcohol, and frequent consumption of mate tea (Ilex paraguariensis; a traditional South American beverage) also didn’t show association with the development of DS when analyzed in a bivariated mode.

Among surgical factors, this complication occurred in single-tooth extraction procedures in all cases, and significantly occurred when odontosection was necessary (Pearson's Chi-square test, *p* =.001) or when the surgical technique involved a combination of flap elevation, osteotomy, and odontosection, indicating a more complex procedure (ordinal-by-ordinal Kendall Tau-B Test, *p* =.002). DS significantly occurred more often when the surgeon also reported a difficult or more complex dental extraction, independently of the technique complexity (Pearson's Chi-square test, *p* <.001) yet, the view of complexity by the surgeon and the technique complexity were associated (ordinal-by-ordinal Kendall Tau-B Test, *p* <.001). In 7.5% [102] of cases, intraoperative accidents or complications occurred, with root fractures being the most frequently reported. Among those cases, two individuals developed DS.

**Table 1:**
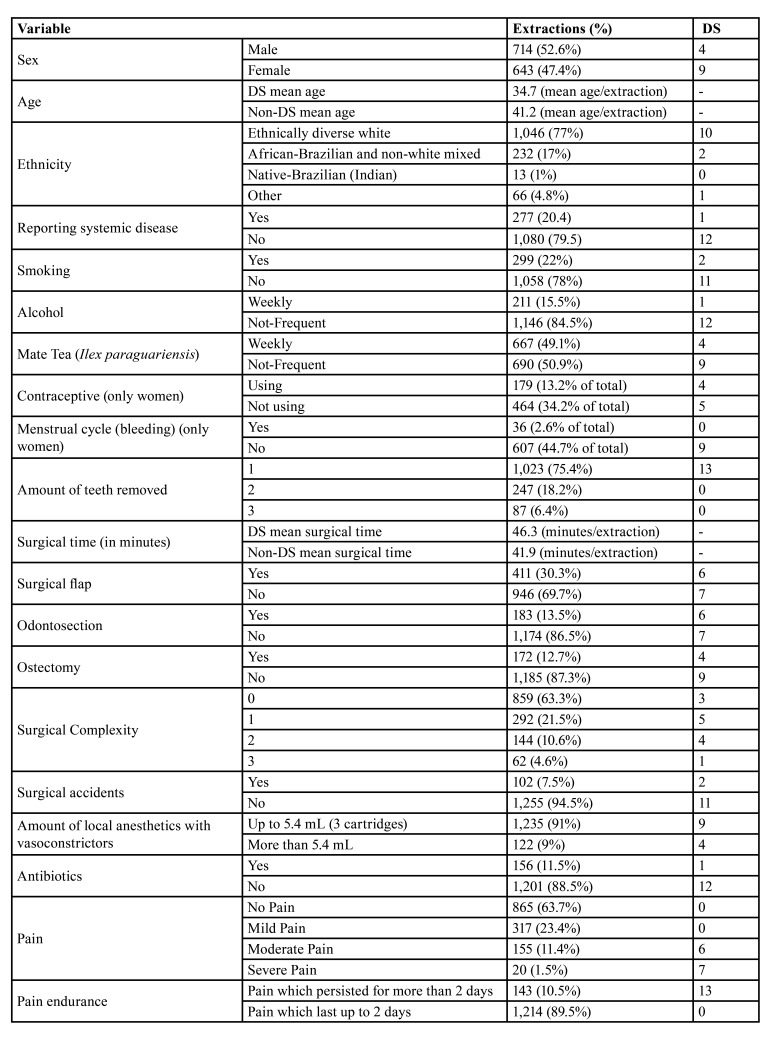
Epidemiological data of the study including the proportions and distributions of dental extractions and the development of dry socket (DS) (n. 1,357).

The duration of the surgery did not significantly influence DS development (NS); however, DS occurred more often in a slightly longer procedures (~42 minutes for cases without DS and ~47 minutes for cases with DS). In terms of the number of anesthetic cartridges used, 61.3% of procedures utilized up to 2 cartridges [832]. When more than 3 cartridges of local anesthetics (>5.4 ml) were used, there was a statistically significant increase in the occurrence of DS (x2, *p* =.005). However, it is important to note that the amount of anesthetics used was also correlated with surgical time and surgical complexity (Spearman's rho Test, rs .26, *p* <.001; rs .18, *p* <.001), which could potentially confound the association between anesthetic dosage and DS incidence.

In this sample, antibiotics were prescribed to 11.5% of cases [156]. Regarding this perioperative prescriptions, it was observed that out of the 13 DS patients, 12 did not receive antibiotics during this phase as part of their regular prescription (unrelated to DS treatment). The use of antibiotics in this sample produced a relative risk reduction for DS of 36% and absolute risk reduction of 0.63%. However, a higher number needed to treat (NNT) indicates that approximately 278 patients would need to be treated with antibiotics to prevent one case of DS. It is important to note that this analysis does not take into consideration the nature of the study, the sample characteristics or the specific criteria for prescribing antibiotics. Antibiotics were significantly more prescribed for older (> 50 years old) patients (x2, *p* <.001), in procedures of greater complexity (Mantel-Haenszel Test for Trend, *p* =.001) and when surgical accidents occurred (x2, *p* =.008).

A Generalized Linear Mediation Model (GLMM) indicated that DS development, only when mediated by surgical complexity, may be influenced by age (GLMM, *p* =.019; younger patients), length of the procedure (GLMM, *p* =.014; longer surgeries) and, surgical accidents (GLMM, *p* =.016; occurrence of it). None of these variables, including surgical complexity and odontosection is related with DS when modulated by the presence of antibiotics. Statistical associations and odds ratios (OR) for DS using bivariable analysis, controlled analysis, or mediated by additional variables are presented in Table 2.

**Table 2:**
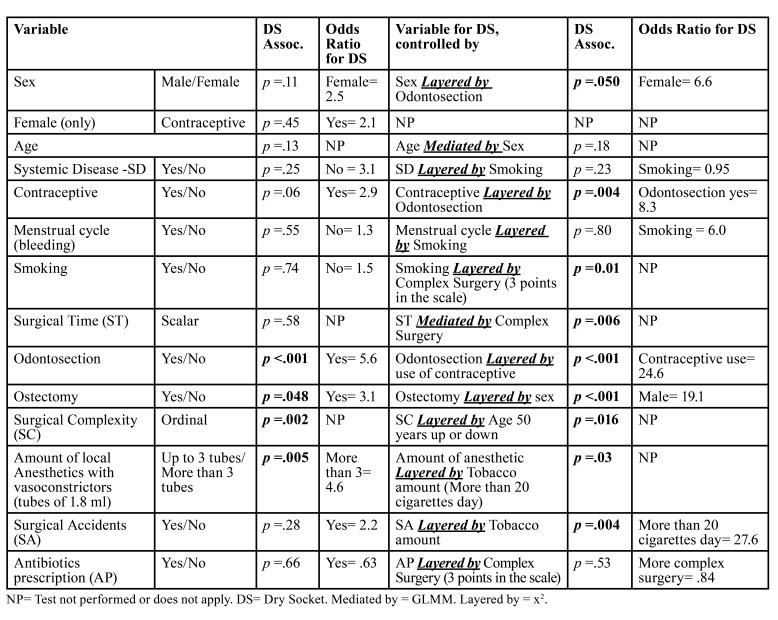
Statistical Associations and Odds Ratios for Dry Socket (DS) using Bivariable Analysis, Controlled (Chi Square Test with Layers, x2), or Mediated (General Linear Mediation Model, GLMM) by Additional Variables (*n*=1,357).

In bivariable analysis, tobacco use as a single variable (Yes/No) did not show an association with DS. However, when analyzing tobacco use as the amount of smoking (more than a pack of 20 cigarettes a day) and considering a more complex surgery, tobacco use was found to be associated with DS development (Mantel-Haenszel Test for Trend, *p* <.015). Additionally, a significant association was observed when surgical accidents occurred and the amount of smoking exceeded a pack (20 cigarettes) per day (Mantel-Haenszel Test for Trend, *p* =.004). These findings suggest that the impact of tobacco use on DS development may be influenced by the quantity of smoking and the complexity of the surgical procedure.

A predictive or warning sign for DS development was the presence of reports of higher pain levels (ordinal-by-ordinal Kendall Tau-B test, *p* =.001), particularly if it persisted for more than two days (Pearson's Chi-square test, *p* <.001). All pain indicators for DS were reported as moderate (6 cases) or high (7 cases), despite the patients' utilization of analgesics.

## Discussion

In the present study, we aimed to investigate the prevalence and associated factors of dry socket (DS) following tooth extractions. DS is a well-recognized and distressing complication that can occur after tooth extraction, causing considerable pain and discomfort for patients. Despite its clinical importance, the pathogenesis of dry socket (DS) remains elusive and probably multifactorial (6), which has hindered the development of effective preventive measures and optimal management strategies.

Although previous studies, such as Oyri *et al*. (4), have reported a higher frequency of DS in female patients compared to males (6:1), the findings from this present study suggest that gender may not be an isolated significant factor in DS development, although it was observed with higher prevalence and odds ratio for women. Furthermore, the present study explored the potential influence of daily contraceptive use on DS development in women and yet it didn’t reach the significant level. Furthermore, the literature does not provide a clear indication or hypothesis regarding the reasons why women are more affected by this condition, while some other authors observes a ratio of 1:1 among man and woman (9). The association between gender and DS remains an intriguing aspect that requires further investigation.

Interestingly, our study findings revealed a significant association between the use of odontosection technique and the development of DS. Specifically, when this surgical procedure was employed, it led to a significantly higher incidence of DS in women, while no such association was observed in men. It is worth noting that DS is intricately linked to the process of fibrinolysis, which involves the degradation of blood clots. In the context of DS, bone exhibits fibrinolytic activity due to the release of plasminogen activators (Pas) triggered by traumatic bone exposure. This complex cascade of inflammatory biochemical events ultimately leads to a decrease in bone formation, increased degradation of blood clots, and delays in wound healing (6). The association between odontosection and the development of dry socket (DS) may be attributed to additional trauma to the surrounding alveolar bone and soft tissues. The increased tissue trauma and manipulation associated with odontosection can lead to a more extensive inflammatory response, delayed healing, and compromised blood clot formation, all of which are known contributing factors to the development of DS.

The association between ostectomy and the development of dry socket (DS) showing a higher incidence in men but not in women, as observed in our study, is an interesting finding that suggests the influence of gender-technique-related factors. Hormonal differences, immune response variations, genetic predisposition, and other variables such as smoking and surgical complexity may contribute to the likelihood of DS development. Further research is needed to elucidate the precise mechanisms underlying these associations.

One important factor that has been extensively discussed in the literature is the impact of smoking on the increased risk of DS (6,8,12,16). Our findings suggest that smoking alone may not be a significant factor in the development of DS. However, our results indicate that smoking, particularly in individuals who consume more than 20 cigarettes a day, becomes a complicating factor when combined with complex surgeries, surgical accidents, and higher amounts of anesthetics used during the procedures. This suggests that the detrimental effects of smoking on DS development may be more pronounced in specific contexts and conditions.

The use of antibiotics to reduce postoperative complications is a subject of frequent discussion in dental literature, particularly in relation to third molar surgeries (7,18). Antibiotics have been proposed as a potential means to decrease the incidence of dry socket (DS), suggesting that specific microbiota may be linked to the pathophysiology of DS, or that antibiotics may act through alternative mechanisms to indirectly prevent blood clot dissolution (1,19). This could potentially involve inhibiting the fibrinolytic properties of certain microorganisms, preventing the dissolution of blood clots and impeding microbial invasion into the wound (6). The hypothesis suggests that bacteria or debris may stimulate the release of cytokines by monocytes/macrophages, which can up-regulate urokinase-type plasminogen activator (uPA) and plasminogen activator inhibitor 1 (PAI-1), ultimately leading to clot lysis (2,3). However, it is important to note that Chow *et al*. (3) argue that current evidence does not support the routine administration of prophylactic antibiotics to prevent DS. Our understandings align with this perspective, as the number needed to treat (NNT) with antibiotics to prevent a single case of DS was exceptionally high. This highlights the need for judicious use of antibiotics and suggests that they should be reserved for specific situations, such as complex surgeries or patients with compromised health conditions. It is essential to consider the potential consequences of antibiotic overuse and misuse, as they contribute to the growing issue of antimicrobial resistance (20). The role of antibiotics in preventing DS and the underlying mechanisms involved require further research and clarification to establish more definitive recommendations in dental practice (5).

As anticipated, the presence of pain emerged as a significant marker for DS development in our study. The severity of pain experienced by patients may be attributed to the upregulation of TNF-α, a pro-inflammatory cytokine known to play a role in the delayed healing process. Studies have shown that elevated levels of TNF-α are associated with reduced bone formation and heightened pain perception, therefore, the intense pain reported by individuals with DS could be attributed, at least in part, to the increased expression of TNF-α and its impact on bone healing processes (6).

The presence of various risk factors associated with DS, such as gender, habits (e.g., smoking), surgical complexity, and the potential role of the microbiota, highlights the multifactorial nature of this inflammatory complication. While the exact etiology or the pathophysiology of DS remains unclear, the findings from this study support the notion that DS is a complex condition influenced by multiple factors.

- Study Limitations

The study may have limitations that should be considered. Firstly, the occurrence of dry socket (DS) was relatively low, which may limit the statistical power and precision of the findings. Additionally, the prescription of antibiotics for some patients could have influenced DS development to some extent. As an observational study, the findings are based on associations rather than causation. Unmeasured confounding factors, such as the use of antiseptics, could potentially have influenced the occurrence of dry socket.

## Conclusions

In conclusion, our study involving 1,357 patients undergoing routine dental extractions revealed a 1% prevalence of dry socket. The obtained results suggests that DS is a multifactorial condition influenced by various factors, including gender, age, smoking, antibiotic prescription and surgical factors such as length, technique and accidents, nevertheless, those associations were observed mainly considering the influence of one variable on another.

## Data Availability

Data are available on request from the Correspondence upon reasonable reason.
